# Genetic Diversity of Circulating Rotavirus Strains in Tanzania Prior to the Introduction of Vaccination

**DOI:** 10.1371/journal.pone.0097562

**Published:** 2014-05-20

**Authors:** Sabrina J. Moyo, Bjørn Blomberg, Kurt Hanevik, Oyvind Kommedal, Kirsti Vainio, Samuel Y. Maselle, Nina Langeland

**Affiliations:** 1 Department of Clinical Science, University of Bergen, Bergen, Norway; 2 Department of Microbiology and Immunology, Muhimbili University of Health and Allied Sciences, Dar es Salaam, Tanzania; 3 Centre for Tropical Infectious Diseases, Haukeland University Hospital, Bergen, Norway; 4 Department of Microbiology, Haukeland University Hospital, Bergen, Norway; 5 Division of Infectious Disease Control, Norwegian Institute of Public Health, Oslo, Norway; 6 Department of Medicine, Haukeland University Hospital, Bergen, Norway; Instituto de Higiene e Medicina Tropical, Portugal

## Abstract

**Background:**

Tanzania currently rolls out vaccination against rotavirus-diarrhea, a major cause of child illness and death. As the vaccine covers a limited number of rotavirus variants, this study describes the molecular epidemiology of rotavirus among children under two years in Dar es Salaam, Tanzania, prior to implementation of vaccination.

**Methods:**

Stool specimens, demographic and clinical information, were collected from 690 children admitted to hospital due to diarrhea (cases) and 545 children without diarrhea (controls) during one year. Controls were inpatient or children attending child health clinics. Rotavirus antigen was detected using ELISA and positive samples were typed by multiplex semi-nested PCR and sequencing.

**Results:**

The prevalence of rotavirus was higher in cases (32.5%) than in controls (7.7%, P<0.001). The most common G genotypes were G1 followed by G8, G12, and G4 in cases and G1, G12 and G8 in controls. The Tanzanian G1 variants displayed 94% similarity with the Rotarix vaccine G1 variant. The commonest P genotypes were P[8], P[4] and P[6], and the commonest G/P combination G1 P[8] (n = 123), G8 P[4] and G12 P[6]. Overall, rotavirus prevalence was higher in cool (23.9%) than hot months (17.1%) of the year (P = 0.012). We also observed significant seasonal variation of G genotypes. Rotavirus was most frequently found in the age group of four to six months. The prevalence of rotavirus in cases was lower in stunted children (28.9%) than in non-stunted children (40.1%, P = 0.003) and lower in HIV-infected (15.4%, 4/26) than in HIV-uninfected children (55.3%, 42/76, P<0.001).

**Conclusion:**

This pre-vaccination study shows predominance of genotype G1 in Tanzania, which is phylogenetically distantly related to the vaccine strains. We confirm the emergence of genotype G8 and G12. Rotavirus infection and circulating genotypes showed seasonal variation. This study also suggests that rotavirus may not be an opportunistic pathogen in children infected with HIV.

## Introduction

Rotavirus is a major cause of severe dehydrating diarrhea both in developed and developing countries [1], [2]. The WHO rotavirus surveillance networks estimates that more than a third of diarrhea hospitalizations among children under five years of age is attributed to rotavirus infection [3]. In 2008, rotavirus caused an estimated 453000 deaths in children younger than five years, more than half of which occurred in developing countries [4].

Due to the limited effect of sanitation-based strategies for preventing the spread of the virus, several rotavirus vaccines have been developed, out of which two oral vaccines (RotaTeq and Rotarix) have been licensed. The introduction of rotavirus vaccines in developed countries has significantly reduced diarrheal mortality and hospitalizations [5]–[10]. A live attenuated monovalent vaccine (Rotarix) was introduced in Tanzania early 2013 and is implemented in the national childhood vaccination schedule.

Rotaviruses are non-enveloped viruses of the genus Reoviridae [11]. The viral genome consists of double stranded segmented RNA enclosed in three protein layers. The middle-layer major capsid protein, VP6 determines the seven major groups (A through G) of rotavirus. Most human infections are caused by group A rotaviruses [12]. The outer capsid of rotavirus is composed of two structural proteins, VP4 (a protease cleaved protein, P protein) and VP7 (a glycoprotein, G protein), which independently elicit protective neutralizing antibodies and serve as the basis of a dual serotyping system [13]–[15]. At least 27 G genotypes and 35 P genotypes have been identified in human rotaviruses [16], whereof genotypes G1P[8], G2P[4], G3P[8], G4P[8], and G9P[8] are responsible for 80–90% of the childhood rotavirus disease burden globally [17]. Rotavirus G12 has recently been identified as an emerging genotype [9]. But from the African continent, G12 in combination with P[6] or P[8] has been reported from only South Africa, Malawi and Nigeria [18]–[20]. Rotavirus G8 genotype has been reported from humans worldwide with high prevalence in some African countries [17], [21]–[28].

Rotavirus infection is common in both temperate and tropical climatic areas and shows distinct seasonality [29]. Before the discovery of the viral agent it was called ‘winter diarrhea and winter gastroenteritis’ in some parts of the world [29]–[31]. There is an increase in attention to the nature of rotavirus disease in relation to seasonality, and this has been well documented in temperate countries [32], [33]. Recently two studies have also demonstrated the inverse relationship between temperature and rotavirus incidence in the tropics [29], [34].

In Tanzania, it is estimated that rotavirus causes more than one third of diarrheal disease hospitalizations and each year it kills more than eight thousands children under five years of age [35]. In a previous study conducted in 2005–2006 [36], we reported high prevalence of rotavirus G9 among under-five children admitted with diarrhea in Dar es Salaam. The present study provides an update on the distribution of rotavirus G and P genotypes among children with diarrhea (cases) and compares it with that from children without diarrhea (controls) between August 2010 and July 2011. In addition we assessed the impact of HIV, clinical features and seasonal variation on rotavirus infection. The findings provide baseline information on rotavirus infection shortly before vaccine introduction in Tanzania.

## Material and Methods

### Ethics Statement

This study received ethical approval from the Senate Research and Publications Committee of Muhimbili University of Health and Allied Sciences in Dar es Salaam, Tanzania and from the Regional Committee for Medical and Health Research Ethics (REK) in Norway. Permission was also obtained from the respective hospital authorities where recruitment of study participants took place (i.e. MNH, Amana and Temeke Hospitals). Written informed consent was obtained from the parent, next of kin, caretaker, or guardian on behalf of all the minors/children enrolled in the study.

### Study Population

This study was conducted between August 2010 and July 2011 in Dar es Salaam Tanzania, a city with a population of about five million. Sample collection was performed during two seasons, starting in August 2010 and in March 2011, aiming for minimum 300 cases and 300 controls in each period. The target for cases was reached in January 2011 and in June 2011, while enrollment of controls continued in February 2011 and July 2011. A total of 1266 stool specimens were collected from children below two years of age. Cases were children hospitalized due to diarrhea at three major hospitals of Dar es Salaam; Muhimbili National Hospital (MNH), Amana and Temeke Municipal Hospitals. MNH, with a bed capacity of 1200, is the largest hospital in the country and serves as a tertiary and national referral Hospital. Amana and Temeke are Municipal district Hospitals of Dar es Salaam. Controls included children below two years of age with no history of diarrhea for one month prior to the study enrollment. These were either children attending child health clinics for immunization and growth monitoring (CHC, n = 310) or children admitted to hospital due to diseases other than diarrhea (n = 235).

### Inclusion Criteria Case Definition

Children admitted in the diarrhea wards with acute or persistent diarrhea were included in the study. Diarrhea was defined as three or more watery stools within 24 hours. An episode of diarrhea was considered over when two consecutive days pass without diarrhea. An episode of acute diarrhea was defined as diarrhea with duration between 24 hours and less than 14 days. Persistent diarrhea was defined as diarrhea for 14 days or more.

Controls included in the study were children without history of diarrhea for one month prior to enrollment. Controls were not matched by age and sex with cases.

### Exclusion Criteria

Children above 24 months of age and cases that could not provide stool sample on the day of admission were not included in the study. Cases and controls whose parent or guardian did not consent to participate in the study were excluded.

### Collection of Information from Children with Diarrhea (Cases) and from Children without Diarrhea (Controls)

Recruitment of cases was done during the admission of the child in the pediatric diarrhea wards of the study sites. A standardized questionnaire was used to collect demographic and clinical information, including age (date of birth), sex, place of residence, parent/guardian level of education and history of antibiotic use prior to admission. Consistency of stool (watery, bloody) and duration of diarrhea was also recorded. The child’s length and weight measurements were recorded. Additional clinical information of patients, such as hydration status, as assessed on the day of admission by the attending clinician, was obtained from patient files together with HIV testing results. HIV testing was done by HIV-DNA PCR at the Hospital laboratory (MNH) or research laboratory at MUHAS, as previously described [37]. Potential controls were enrolled during the recruitment of cases. The questionnaire for controls did not have parameters for clinical characteristics such as type of diarrhea and presence of dehydration. In addition controls did not have diarrhea for one month prior to the study enrollment.

### Weight and Length Measurements

All children were weighed using a 25 kg Salter hanging scales (CMS Weighing equipment, High Holborn, London, United Kingdom). Weight was recorded to the nearest 0.1 kilogram. Length was measured using standard length boards to the nearest millimeter. Weight for age (WAZ), length for age (LAZ) and weight for length (WLZ) Z-scores were calculated using EPI Info (USD, Inc., Stone Mountain, GA). Children were categorized to have normal nutritional status, mild or severe malnutrition using Z-scores according to WHO criteria [38].

### Specimen Collection

A single stool specimen was collected on inclusion from each child using wide mouthed sterile plastic containers. One portion was frozen at −70°C the same day as it was collected.

### Rotavirus Detection

Rotavirus antigens were detected using the commercially available ProSpecT Rotavirus ELISA kit (Oxoid, Hants, UK), with 10% fecal suspensions according to the manufacturer’s instructions.

### Extraction of RNA

Fifty µg of rotavirus antigen-positive stool specimen were mixed 1∶10 with Bacterial Lysis buffer (Roche Applied Science, Mannheim, Germany), and centrifuged at 13.000 g for 3 minutes. RNA was extracted from 200 µL supernatant using the Magna Pure LC High Performance Total Nucleic Acid Isolation Kit (Roche Applied Science, Mannheim, Germany).

### Reverse Transcription, G and P-typing of Rotaviruses

Reverse transcription, G and P typing was done as described in European Rotavirus Detection and Typing Methods version 4 [39]. Briefly a total of 40 µL of RNA extract was used as template for reverse transcription with random primers. Rotavirus G an P genotyping was performed using semi-nested type specific multiplex PCR's that could detect eight G genotypes (G1, G2, G3, G4, G8, G9, G10 and G12) and six P-types P[4], P[6], P[8], P[9], P[10] and P[11]. PCR products were subjected to electrophoresis on a 2% agarose gel, stained with Gel Red, and observed under ultraviolet light. The Rotavirus G and P genotypes were determined by their specific size on the agarose gel. The non-typeable rotavirus strains were further confirmed if they were rotavirus using a single round rotavirus VP6 specific PCR. Negative and positive controls were included in all PCR assays.

### Sequencing of the VP7 and VP4 Gene of the Rotavirus Positive Strains

Partial sequencing of the first round PCR-amplicons for the VP7 gene was performed for all samples positive for rotavirus in order to confirm the PCR G- typing results as described in the European Rotavirus Detection and Typing Methods version 4 [39]. Randomly selected rotavirus positive isolates (33%) were subjected to partial sequencing of the VP4 gene to confirm PCR P-typing results. The sequencing of both VP4 and VP7 was done using purified first round PCR products, on an ABI3730 machine using BigDye (Applied Biosystems). The same primers as in the PCR were used, both forward and reverse primers for VP7 gene while only forward primers was used for VP4 gene.

### Sequence and Phylogenetic Analysis of the VP7 Gene

Nucleotide sequences were analyzed using the RipSeq interpretation software (iSentio Ltd., Bergen, Norway) and by the nucleotide BLAST service (NCBI). The evolutionary distances between Tanzanian strains, vaccine strains and the reference strains from GenBank were investigated using pairwise comparison from multiple sequence alignments using the Genius software package (Biomatters) and the phylogenetic tree was constructed using the UPGMA and Kimura two-parameter methods [40]. A bootstrap resampling analysis was performed (1000 replicates) to test tree reliability.

### Nucleotide Sequence Accession Numbers

The DNA sequences for the VP7 genes of the study strains were submitted directly to GenBank and were assigned accession numbers from KF976838 to KF976860.

The DNA sequences for the VP4 genes of the study strains were submitted directly to GenBank and were assigned accession numbers from KF976815 to KF976837.

### Statistical Analysis

Weight for age, length for age and weight for length Z-scores were calculated using EPI Info (USD, Inc., Stone Mountain, GA, USA). Statistical analysis was performed using the Statistical Package for the Social Sciences (SPSS for IBM-PC, release 18.0; SPSS Inc., Chicago, IL, USA). Differences in proportions were tested using the chi-square (**χ** ^2^) test. A p-value of <0.05 was considered significant. The association between rotavirus positivity, genotypes, clinical and demographic characteristics was estimated as the odds ratio (OR) between rotavirus infected and rotavirus uninfected individuals in a logistic regression model. Factors from the univariate analysis were kept in the main logistic regression model. A separate regression model including HIV as a factor, employed manual, backwards, stepwise elimination of non-significant factors (p≥0.05).

## Results

During the study period August 2010 to July 2011, a total of 1266 samples were collected. Among 1235 samples containing adequate material for rotavirus detection, 690 were from children with diarrhea and 545 were from controls (children without diarrhea). There were 717 (58.1%) males and 517(41.9%) females. Children were recruited from the districts Ilala (n = 634, 51.3%), Kinondoni (n = 360, 29.1%) and Temeke (n = 241, 19.5%).

### Prevalence of Rotavirus Infection and Distribution of G and P Genotypes

Of the 1235 children with and without diarrhea included in the study, rotavirus was detected in 266 children (21.5%) using ELISA. The prevalence of rotavirus infection was significantly higher in cases (32.5%, 224/690) than in controls (7.7%, 42/545, P<0.01, OR = 5.8, 95%CI 4.045 to 8.192). The prevalence of rotavirus did not differ statistically between controls from Child Health Clinics (7.7%, 24/310) and hospital based controls (7.7%, 18/235, P>0.05).

Sequencing results detected rotavirus G8 in 33 cases, which were misallocated to G12 when G genotype was assessed by PCR alone. Therefore, all samples were G typed based on sequencing results. A total of 211 specimens, which were successfully sequenced were characterized to G genotypes, 190 cases and 21 controls. Out of eight rotavirus G genotypes searched for, only five G genotypes were found. The most commonly detected G genotype strains among cases were G1 (n = 131, 68.6%), followed by G8 (n = 33, 17.3%), G12 (n = 21, 11.05%), G4 (n = 4, 2.1%) and G9 (n = 1, 0.5%). Rotavirus genotype G1 was also common among children without diarrhea (controls) accounting for 66.7% followed by G12 (19.0%) and G8 (14.3%). Genotype G2, G3 and G10 were not detected.

A total of 236 samples were P genotyped using RT-PCR, 211 were cases and 25 were controls. One third of 236 samples underwent sequencing of the VP4 gene, which produced the same results as RT-PCR. Out of six P genotypes searched for, only three P genotypes were found. The commonest circulating P genotype in cases was P[8] (n = 141, 66.8%) followed by P[4] (n = 40, 19.0%) and P[6] (n = 30, 14.2%). Rotavirus P[9], P[10]and P[11] were not found. Table 1 shows G/P combinations for the 211 samples that were successfully typed for both G and P genotypes (among the 236 P-typed samples, 25 could not be G-typed). The commonest G/P combination in cases was G1P[8] accounting for 123 samples, followed by G8P[4] (n = 27) and G12P[6] (n = 21).

**Table 1:** Sequencing results of rotavirus G and P genotypes circulating in children with diarrhea (cases) and children without diarrhea (controls) in Dar es Salaam, Tanzania (n = 211; 190 cases and 21 controls).

	G1	G4	G8	G9	G12	Total P types N (%)
**Cases**						
P4	7	2	27	0	0	36 (19.0%)
P6	1	2	6	0	21	30 (15.7%)
P8	123	0	0	1	0	124 (65.4%)
**Total G types**	131 (68.6%)	4 (2.1%)	33 (17.3%)	1 (0.5%)	22 (11.5%)	**190** (100%)
**Controls**						
P4	0	0	3	0	0	3 (14.3%)
P6	0	0	0	0	4	4 (19.0%)
P8	14	0	0	0	0	14 (66.7%)
**Total G types**	14 (66.7%)	0	3 (14.3%)	0	4 (19.0%)	**21** (100%)

We found no rotavirus G or P genotype associated with diarrhea. The common G and P genotypes circulating in children with diarrhea were also common among children without diarrhea as shown in Table 1. We did not find samples with multiple strains of rotavirus.

### Analysis of VP7 Nucleotide Sequences

**Figure 1:**
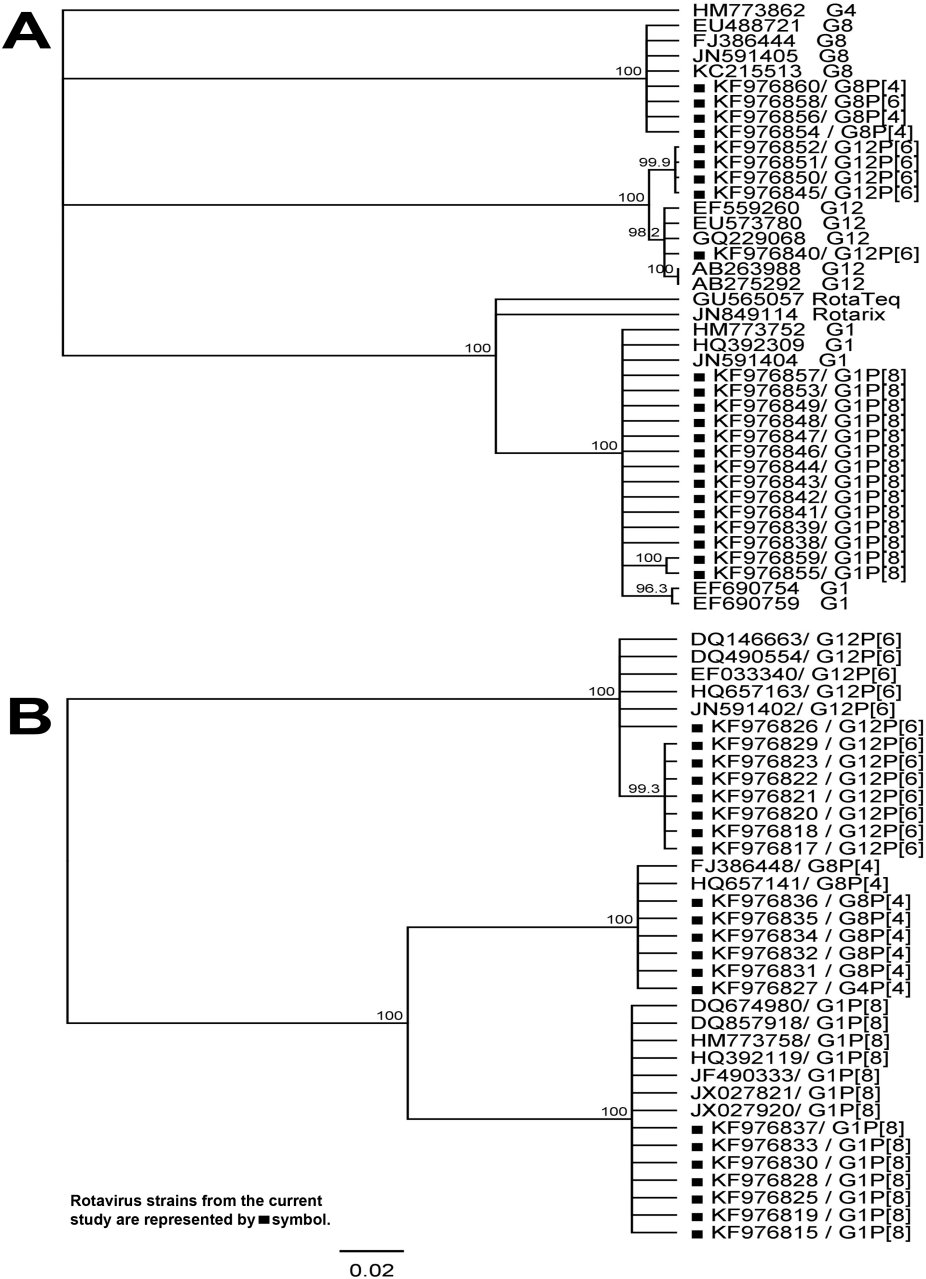
A and B. Phylogenetic trees of the rotavirus nucleotide sequence of the partial VP7 and VP4 genes. The phylogenetic tree: Phylogenetic trees based on the nucleotide sequence of the partial VP7 gene (figure 1A) and VP4 gene (figure 1B) of rotaviruses from Tanzania with known rotavirus reference strains from GenBank database and rotavirus vaccine strains i.e Rotateq and Rotarix. Reference strains, vaccine strains and strains from this study are indicated by accession numbers. The Genius software package was used to build the tree with the UPGMA method and bootstrapped with 1,000 repetitions; The Kimura-2 substitution model was used. The bar indicates nucleotide substitutions per site.

Phylogenetic analysis based on VP7 nucleotide sequences showed that sequences varied by only 1–2% within the rotavirus G1 strains detected in Tanzania (Fig. 1A). These GI genotypes also showed 98–99% nucleotide similarity to G1 strains circulating worldwide such as in Malawi, Bangladesh, Belgium and USA with GenBank accession numbers JN591404, EF690754, EF690759, HQ392309 and HM773752. Notably, only 94% similarity was found between the Tanzanian G1genotype and the rotavirus vaccine-strains RotaTeq (GU565057) and Rotarix (JN849114).

The rotavirus G8 strains in this study had 98–99% nucleotide similarity with each other; they are also closely related (98%–99% nucleotide similarity) to circulating rotavirus G8 strains in Kenya, Malawi and USA with GenBank accession numbers EU488721, FJ386444, JN591405, GQ496281, JF693231, KC215513. The G12 genotypes in this study displayed nucleotide similarities above 99%. These rotavirus G12 strains were also closely related to circulating G12 strains in Malawi, India and Nepal with GenBank accession numbers EU573780, EF559260, AB263988 and AB275292.

### Analysis of VP4 Nucleotide Sequences

Comparison of VP4 nucleotide sequences from Tanzanian strains and representatives of P-genotype strains from the GenBank database are shown in figure 1B. Tanzanian P[8] strains showed 98% nucleotide identity to each other and to circulating P[8] reference strains from Australia, Brazil, USA and Thailand with GenBank accession numbers JF490333, JX027920, DQ857918, HM773758 and DQ674980. Nucleotide identities between Tanzanian rotavirus P[4] strains and reference P[4] strains from Malawi and Kenya was 98%. GenBank accession numbers for P[4] reference strains are FJ386448 and HQ657141. Tanzanian rotavirus P[6] strains were closely related to each other with 99% nucleotide similarity and showed 98% nucleotide identity to reference P[6] strains from Malawi, South Africa and Bangladesh with GenBank accession numbers JN591402, HQ657163, EF033340, DQ490554, DQ146663.

### Seasonality of Rotavirus Infection and Rotavirus G-types

The prevalence of rotavirus detected varied significantly by months in both cases and controls (P<0.001). When results were divided into cool and hot months of the year i.e. May through August vs. November through February, rotavirus prevalence (both in cases and controls) was significantly higher in cool than hot months (23.9% vs. 17.1%, P = 0.012, OR 1.52, 95% CI: 1.081 to 1.894: 1.09 to 2.11). As shown in Figure 1, there was also high number of rotavirus detected among cases in the beginning of the cool season i.e. April 2011 and between the cool and the hot season, which are periods that coincide with rain season.

**Figure 2:**
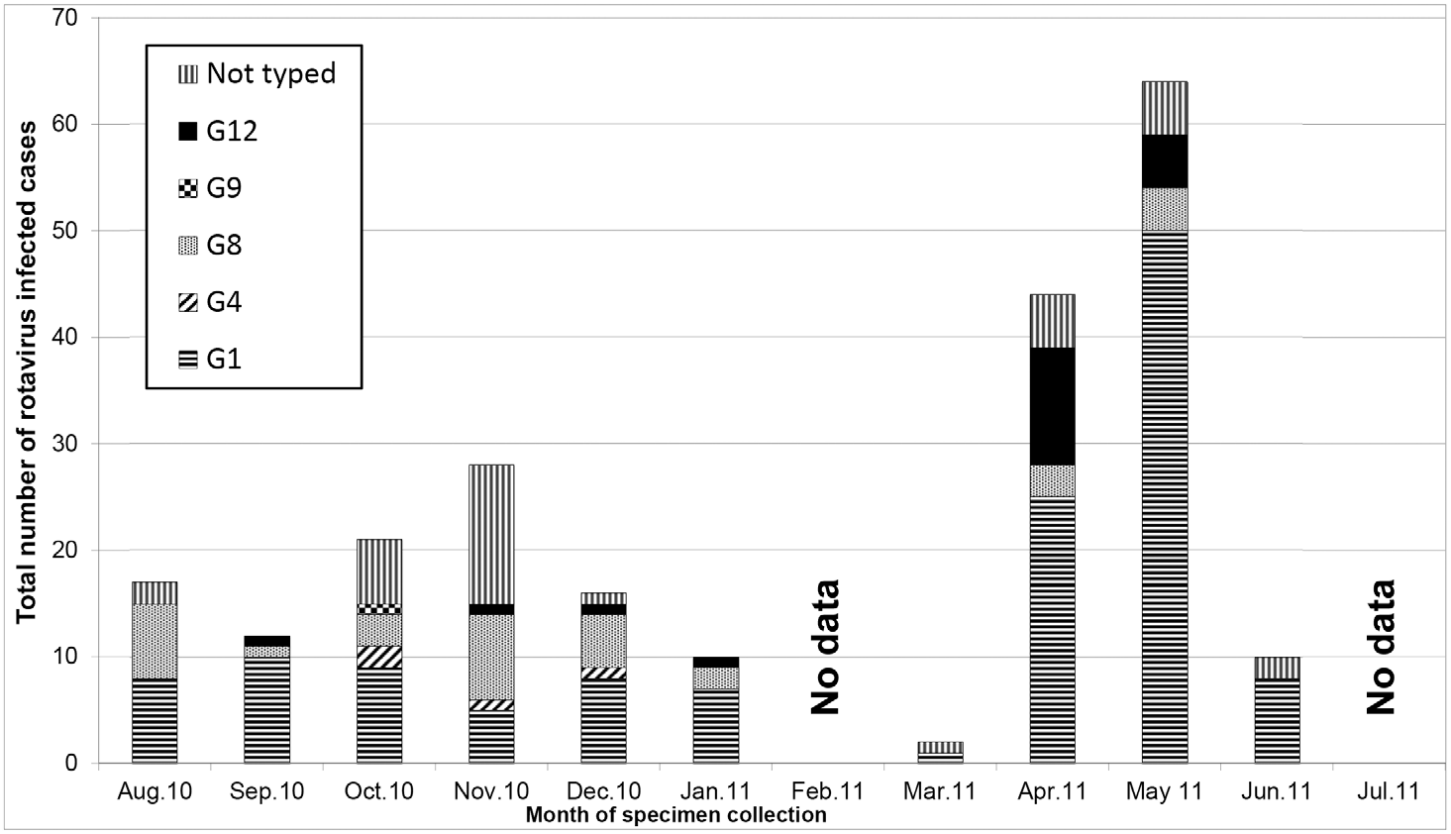
Seasonal variation of rotavirus infection and G genotypes among children admitted with diarrhea. The graph shows the total number of rotavirus infected children admitted due to diarrhea (cases) per month with G genotypes.

We also observed significant variations of G genotypes detection in different months during the study period (P<0.001) as shown in Figure 2. The commonest genotype G1 was detected in most months of the study, with the highest peak in the month of April 2011, which is the beginning of the cool season and May 2011. Genotypes G8 and G12 were detected in most months of the study period, but genotype G9 was only detected in the month of October 2010.

We compared rotavirus prevalence in studies conducted in previous years in the same region but during different seasons of the year. We observed high prevalence in studies conducted during the cool months of the year [41], [42] compared to studies conducted in hot months of the year, Table 2 [36], [43].

**Table 2:** Summary of studies on rotavirus in Dar es Salaam, Tanzania.

	Brookfield *et al* 1979[41]	Mhalu *et al* 1988[43]	Sam *et al* 1992[42]	Moyo *et al* 2007[36]	Current study 2014
Prevalence of rotavirus	31%	19.5%	43%	18.1%	32.5%
Type of subjects	Inpatients	Inpatients	Inpatients	Inpatients	Inpatients
Months of data collection	Mar1976–Sept 1976	Jan 1987–Feb 1987	May 1988–Aug 1988	Dec 2005–Feb 2006	Aug 2010–Jul 2011
Age group studied	<7 yrs	<3 yrs	<3 yrs	<5 yrs	<2 yrs
Method for detection	EM	Latex agglutination	EM+ latex agglutination	ELISA+PCR	ELISA+PCR

### Association between Rotavirus Infection and HIV Status

A total of 421 children were tested for HIV, of these 33 and 388 tested HIV positive and negative respectively. In univariate analysis, the prevalence of rotavirus infection was significantly lower in HIV-infected (15.4%, 4/26) than in HIV-uninfected children with diarrhea (cases) (55.3%, 42/76, P = 0.001, OR 0.152, 95% CI: 0.05–0.48). HIV status was not included in the final regression model; because it would introduce a high number of missing values as 54% rotavirus infected children were not tested for HIV. A separate regression model was performed including HIV status and other significant risk factors from the univariate analysis (length for age, place of residence and type of diarrhea). With stepwise, backwards removal of all non-significant factors (P≥0.05), rotavirus infection remained significantly negatively associated with HIV infection (P = 0.027, OR 0.26, 95% CI: 0.08 to 0.85) and stunting (P = 0.005, OR 0.23, 95% CI: 0.08 to 0.63).

Among children without diarrhea (controls), the prevalence of rotavirus was significantly higher in HIV-infected (28.6%, 2/7) than in HIV-uninfected children (7.4%, 23/312, P = 0.039, OR 5.03, 95%CI: 0.92 to 27.35).

### Distribution of Rotavirus Infection and G/P Genotypes by Age and Sex

**Figure 3:**
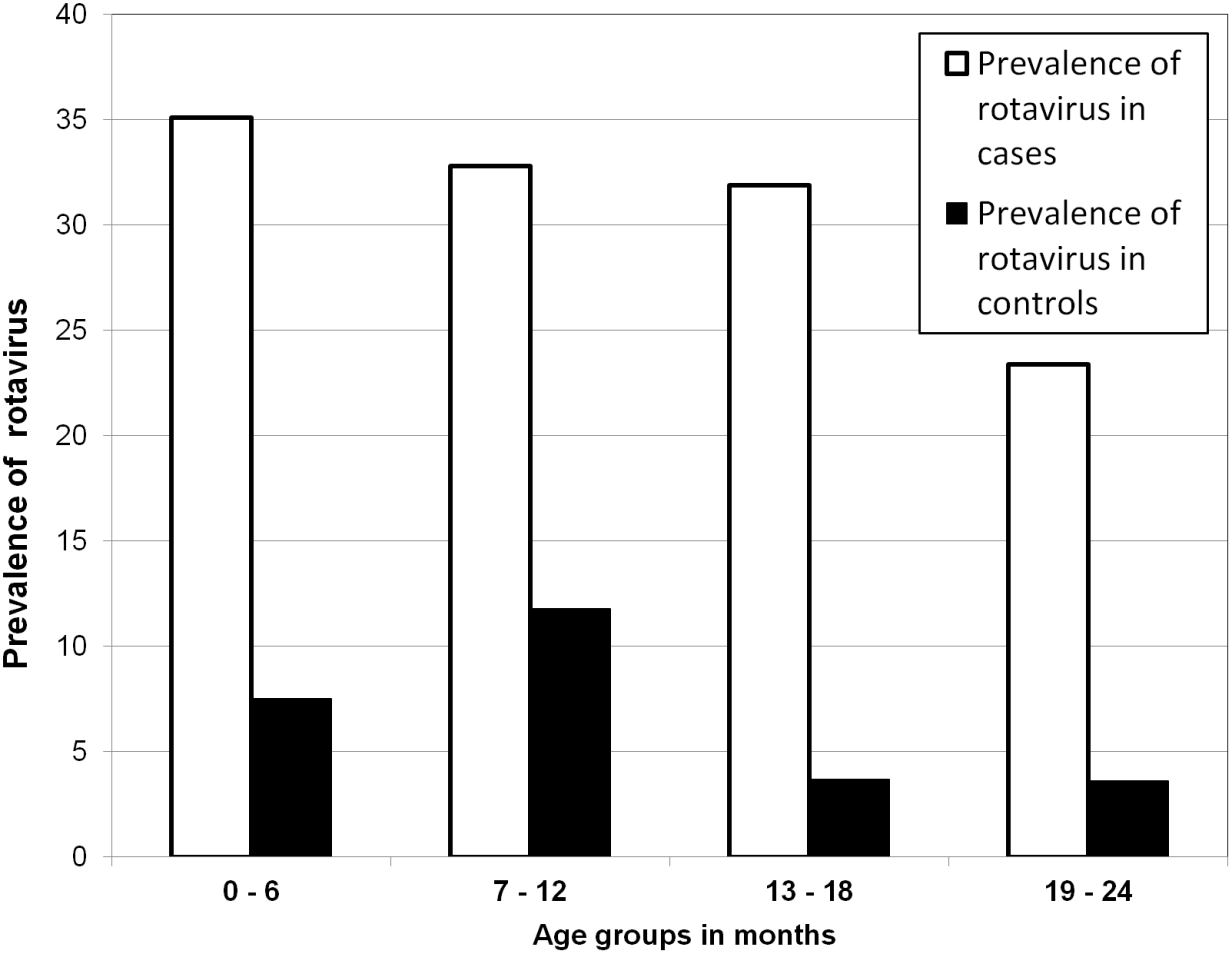
Prevalence of rotavirus infection in different age groups. The graph shows the prevalence of rotavirus from ELISA results per age group in cases and controls.

The median age of all rotavirus infected children (9.6 months) was significantly lower than that of rotavirus uninfected children (10.7 months, P = 0.003). There was no significant difference in the median age of rotavirus infected children with and without diarrhea (9.6 months vs. 9.7 months). Figure 3 shows that the prevalence of rotavirus infection in cases was significantly higher in the age group of 0–6 months compared to the age group ≥19 months (35.1% vs. 23.4%, P = 0.028, OR 2.14, 95% CI: 1.09 to 4.23). The prevalence of rotavirus infection did not differ significantly between age groups 7–12 and 13–18 months respectively, as compared to age group ≥19 months. The prevalence of rotavirus infection in controls did not differ significantly between age groups (P>0.05). Rotavirus genotypes G1 and G8 were detected in all age groups, whereas G4 was detected in the age of 0–12 months and one strain of G9 was detected from a child in the group of 0–6 months.

The proportion of rotavirus infection in all children with and without diarrhea did not differ significantly by sex as shown in Table 3.

**Table 3:** Association between demographic/clinical characteristics and rotavirus infection among cases and controls in Dar es Salaam Tanzania.

				Rotavirus infection					
				Cases			Controls		
		N	N	*Univariate*		*Multivariate^A^*	*Univariate*		*Multivariate^A^*
Demographic/clinical characteristic		Cases	Controls	n (%)	OR (95%CI)	OR (95%CI)	n (%)	OR (95%CI)	OR (95%CI)
**Sex**	Male	422	296	140 (33.1)	1.09 (0.78–1.51)	1.06 (0.75–1.49)	26 (8.8)	1.40 (0.74–2.68)	1.58 (0.81–3.07)
	Female	268	249	84 (31.3)	1	1	16 (6.4)	1	
**Parent/guardian level of education**									
	Primary education	531	412	180 (34.0)	0.72 (0.47–1.10)	0.71 (0.46–1.11)	30 (7.2)	1.32 (0.66–2.67)	1.29 (0.62–2.66)
	Secondary education	137	128	37 (27.0)	0.91 (0.36–2.27)	1.05 (0.40–2.72)	12 (9.4)	0.0 (0.0)	0.0 (0.0)
	Higher education	22	5	7 (3.18)	1	1	0 (0.0)	1	1
**Place of residence (district)**									
	Temeke	150	91	65 (43.3)	0.61 (0.41–0.91)	0.49 (0.32–0.74)**	7 (7.7)	1.15 (0.48–2.75)	1.05 (0.43–2.56)
	Ilala	338	296	108 (32.0)	0.44 (0.28–0.70)	0.44 (0.28–0.71)**	26 (8.8)	0.72 (0.23–2.00)	0.82 (0.29–2.34)
	Kinondoni	201	158	51 (25.4)	1	1	9 (5.7)	1	1
**Type of diarrhea**									
	Acute diarrhea	613	na	212 (34.7)	2.87 (1.52–5.43)	2.81 (1.45–5.46)	na	na	na
	Persistent diarrhea	76	na	12 (15.8)	1	1	na	na	na
**Hydration status on admission**									
	Severe dehydration	157	na	46 (28.9)	1.42 (0.95–2.13)	1.23 (0.84–1.97)	na	na	na
	Some dehydration	362	na	132 (36.8)	0.91 (0.56–1.48)	0.92 (0.55–1.54)	na	na	na
	No dehydration	170	na	46 (27.1)	1	1	na	na	na
**Nutrition status of the child**									
	***i)Underweight (WAZ)***								
	Normal	300	326	107 (35.8)	1	1	30 (9.1)	1	1
	Malnourished	390	219	117 (30.0)	1.30(0.95–1.80)	1.01 (0.68–1.51)*	12 (5.5)	1.74 (0.87–3.47)	1.58 (0.65–3.87)
	***ii)Stunting (LAZ)***								
	Normal	223	242	89 (40.1)	1	1	21 (8.6)	1	1
	Malnourished	190	303	135 (28.9)	0.61(0.43–0.85)	0.61 (0.40–0.94)	21 (6.9)	1.40 (0.75–2.63)	1.07 (0.52–2.21)
	***iii)Wasting(WLZ)***								
	Normal	490	434	158 (32.2)	1	1	34 (7.8)	1	1
	Malnourished	200	111	66 (33.0)	0.96(0.68–1.36)	0.86 (0.55–1.35)	8 (7.2)	1.10 (0.50–2.45)	0.71 (0.27–1.88)

Note: na = not applicable for controls; N = total number of samples tested; n = number of positive rotavirus samples; A = multivariate logistic regression analysis included all variables in the table and age group in months;

* = P<0.05;

** = P<0.01.

The distribution of rotavirus G and P genotypes was not significantly associated with sex of the child, presence of dehydration, nutritional status or HIV status (P>0.05).

## Discussion

Rotavirus is the leading cause of severe diarrhea both in developed and developing countries. This one-year surveillance study described the molecular epidemiology of rotavirus infection in children in Dar es Salaam, the major city of Tanzania with a population of about five million inhabitants. Children with diarrhea were six times more likely to be infected with rotavirus than those without diarrhea. The study confirms findings from other studies twenty years ago in the same location and elsewhere [42], [44] indicating that rotavirus is still a major pathogen causing diarrhea in children in Tanzania. The presence of rotavirus among controls may represent reservoirs for transmission in the community.

In the current study, five G and three P genotypes were detected. Rotavirus G1[P8] was the most prevalent G/P combination and this genotype combination is reported to be responsible for 50–65% of rotavirus infections in children worldwide [45]. Since more than 60% of the study subjects were affected by this genotype combination, which is in the current vaccine introduced in Tanzania (Rotarix), we assume that the vaccine will be protective, given that the circulating genotype is stable. Rotavirus G8 was the second most common genotype in this study and this is the first time it is reported in Tanzania. Of note is the fact that all the G8 strains detected were initially genotyped as G12 by multiplex and semi-nested PCR using primers described in the European Rotavirus Detection and Typing Methods version 4 [39]. The G8 genotype-specific primers were compared to nucleotide sequences of the G8 viruses isolated in this study, and three to four primer mismatches were found predominantly in the 3′end. When G12 genotype-specific primers were compared to G8 sequences we found that the numbers of mismatches were fewer and in less critical positions than the unintended mismatches for the G8 specific primers. Consequently, in samples containing G8 genotype viruses, none of the primers had perfect match, and the G12 primers by chance obtained the strongest binding producing a false positive G12 result. Due to the higher mutation-rates in viral genomes, PCR based typing strategies will generally be more error-prone than typing based on sequencing. This can result in erroneous epidemiological data and a poor foundation for further vaccine research. Other studies have also documented mistyping of rotavirus strains by multiplex RT-PCR [20], [46]–[48]. We suggest that caution should be taken when interpreting the results of rotavirus G genotypes based on multiplex PCR. Furthermore these findings emphasize the higher robustness obtained by sequencing for typing of rotaviruses.

Genotype G12 was previously documented by sequencing only in three African countries, namely Malawi, South Africa and Nigeria [18]–[20]. The present study is the first documentation of genotype G12 in Tanzania.

Surprisingly we found only one strain of G9 in combination with P[8] in the current study, while this genotype predominated in the same study setting 8 years earlier [36]. This emphasizes the need for continuous rotavirus strain surveillance.

Apart from defining the serotype of rotavirus, G proteins are critical to vaccine development because they are targets for neutralizing antibodies that are believed to be important for protection. The two current rotavirus vaccines i.e RotaTeq (RV5) and Rotarix (RV1) can control infection against the five main rotavirus genotypes, which are G1, G2, G3, G4 and G9 [49]. Phylogenetic analysis was performed to show the relationship between the Tanzanian G1P[8] and vaccine strains in the Rotarix and RotaTeq vaccines. Our results revealed that the Tanzanian G1P[8] strains are distantly related to G1P[8] of the vaccine strains. This suggests that circulating G1P[8] strains may have changed over time through accumulated mutations making them different from original vaccine strains which were isolated over twenty six years ago [49], [50]. In this study we report a significant increase in the prevalence of P[6] and P[4] rotavirus positive samples compared to the previous study in the same region [36]. Rotavirus samples with P[6] in this study were associated with a variety of G-genotypes (G4, G8, and G12). Phylogenetic analysis revealed that rotavirus P[6] in this study is closely related to P[6] from other African countries such as Malawi and South Africa. The high prevalence of rotavirus genotypes not included in the current rotavirus vaccines i.e. G8, G12, P[4] and P[6] in this study and other studies from developing countries may be one of the reasons for the reported lower vaccine efficacy in developing countries [51]. The Tanzanian G1P[8] variant may be able to escape from vaccine induced immunity [50]. However other possible contributing factors such as maternal antibodies and change in gut microbiota needs to be investigated [52], [53]. No multiple rotavirus infection of G or P genotypes was detected in this study, which concurs with the previous findings in the same study setting [36]. In this study, rotavirus showed significant variation of prevalence in different months of the year with a peak of rotavirus infection during the cooler months of the year. The seasonal variation observed in the current study is further supported by findings from previous studies from the same region [36], [41]–[43]. Studies conducted during cool months of the year [41], [42] found high prevalence of rotavirus compared to studies conducted during hot months of the year [36], [43]. Understanding seasonal patterns of rotavirus will be useful when considering the appropriate timing of immunization booster programs in settings, which have reported poor efficacy of rotavirus vaccine and have demonstrated strong seasonality. Rotavirus vaccine administration in the current Extended Program on Immunization schedule in developing countries is at 6, 10, and 14 weeks of age. However, if booster vaccination programs were to be considered for older children lacking immunity, vaccination during the pre-rotavirus season is recommended [34].

In this study we found significantly lower incidence of rotavirus in HIV-positive children compared to HIV-negative children with diarrhea. This supports findings of previous studies in Tanzania and other developing countries where rotavirus is detected less.

frequently or not detected at all among HIV-positive children with diarrhea [54]–[59]. On the other hand rotavirus was more prevalent among HIV-positive children than HIV-negative children without diarrhea. More studies are needed to clarify this issue.

Rotavirus diarrhea occurs at an earlier age among children in developing countries than children in industrialized countries. The mean age of rotavirus infection in this study compares with the mean age of rotavirus gastroenteritis in other developing countries which ranges from 6–9 months [60]. However the rotavirus prevalence of 27.8% in children less than three months is notably high with non-vaccine serotypes also found i.e. G8 and G12. This may have implications for the rotavirus vaccine introduced in the study setting; first, this age group may not fully benefit from the immunization programme, since the first dose is given at 6 weeks; secondly these infants may have acquired immunity from natural infection with rotavirus prior to immunization and therefore the ability to measure vaccine efficacy in the study setting may be impaired.

## Conclusion

This study showed a switch from G9 genotype during the past 8 years to G1genotype dominance in this study, and a low similarity between the Tanzanian G1 genotype and the vaccine G1genotype. We have also observed unusual circulating genotypes G8 and G12 for the first time in Tanzania. Since early 2013, the rotavirus vaccine Rotarix has been introduced and included in the Extended Program of Immunization (EPI) in Tanzania. The present study represents pre-vaccination data and may be useful in the future when assessing the effectiveness of the vaccine. This study also showed seasonal variation in the prevalence of rotavirus. Rotavirus seasonality provides insights important for vaccination strategies, including potential shifts in seasonal peaks and duration of outbreaks. Our data also support the notion that rotavirus may not be an opportunistic pathogen in children infected with HIV.

## References

[1] Parashar UD, Burton A, Lanata C, Boschi-Pinto C, Shibuya K, et al (2009) Global mortality associated with rotavirus disease among children in 2004. J Infect Dis 200 Suppl 1S9–S15. 19817620 10.1086/605025

[2] Parashar UD, Gibson CJ, Bresee JS, Glass RI (2006) Rotavirus and severe childhood diarrhea. Emerg Infect Dis 12: 304–306. 16494759 10.3201/eid1202.050006 PMC3373114

[3] Centers for Disease Control and Prevention (2011) Rotavirus surveillance–worldwide, 2009. MMWR Morb Mortal Wkly Rep 60: 514–516. 21527889

[4] Tate JE, Burton AH, Boschi-Pinto C, Steele AD, Duque J, et al (2012) 2008 estimate of worldwide rotavirus-associated mortality in children younger than 5 years before the introduction of universal rotavirus vaccination programmes: a systematic review and meta-analysis. Lancet Infect Dis 12: 136–141. 22030330 10.1016/S1473-3099(11)70253-5

[5] Wang FT, Mast TC, Glass RJ, Loughlin J, Seeger JD (2010) Effectiveness of the pentavalent rotavirus vaccine in preventing gastroenteritis in the United States. Pediatrics 125: e208–213. 20100757 10.1542/peds.2009-1246

[6] de Palma O, Cruz L, Ramos H, de Baires A, Villatoro N, et al (2010) Effectiveness of rotavirus vaccination against childhood diarrhoea in El Salvador: case-control study. BMJ 340: c2825. 20551120 10.1136/bmj.c2825 PMC2886195

[7] Staat MA, Rice MA, Donauer S, Payne DC, Bresee JS, et al (2010) Estimating the rotavirus hospitalization disease burden and trends, using capture-recapture methods. Pediatr Infect Dis J 29: 1083–1086. 21155173 10.1097/inf.0b013e3181fb8f7b

[8] Paulke-Korinek M, Rendi-Wagner P, Kundi M, Kronik R, Kollaritsch H (2010) Universal mass vaccination against rotavirus gastroenteritis: impact on hospitalization rates in austrian children. Pediatr Infect Dis J 29: 319–323. 19935446 10.1097/INF.0b013e3181c18434

[9] Trimis G, Koutsoumbari I, Kottaridi C, Palaiologou N, Assimakopoulou E, et al (2011) Hospital-based surveillance of rotavirus gastroenteritis in the era of limited vaccine uptake through the private sector. Vaccine 29: 7292–7295. 21816195 10.1016/j.vaccine.2011.07.092

[10] Becker-Dreps S, Paniagua M, Dominik R, Cao H, Shah NK, et al (2011) Changes in childhood diarrhea incidence in nicaragua following 3 years of universal infant rotavirus immunization. Pediatr Infect Dis J 30: 243–247. 20881511 10.1097/INF.0b013e3181f87ffe PMC3039066

[11] Martella V, Banyai K, Matthijnssens J, Buonavoglia C, Ciarlet M (2010) Zoonotic aspects of rotaviruses. Vet Microbiol 140: 246–255. 19781872 10.1016/j.vetmic.2009.08.028

[12] Kapikian AZ, Hoshino Y, Chanock RM (2001) Rotaviruses. In: Howley PM, editor. Fields virology 4th ed. Philadelphia, Pa.: Lippincott Williams & Wilkins. 1787–1833.

[13] Hoshino Y, Sereno MM, Midthun K, Flores J, Kapikian AZ, et al (1985) Independent segregation of two antigenic specificities (VP3 and VP7) involved in neutralization of rotavirus infectivity. Proc Natl Acad Sci U S A 82: 8701–8704. 3001716 10.1073/pnas.82.24.8701 PMC391504

[14] Offit PA, Clark HF, Blavat G, Greenberg HB (1986) Reassortant rotaviruses containing structural proteins vp3 and vp7 from different parents induce antibodies protective against each parental serotype. J Virol 60: 491–496. 3021983 10.1128/jvi.60.2.491-496.1986 PMC288917

[15] Laird AR, Gentsch JR, Nakagomi T, Nakagomi O, Glass RI (2003) Characterization of serotype G9 rotavirus strains isolated in the United States and India from 1993 to 2001. J Clin Microbiol 41: 3100–3111. 12843049 10.1128/JCM.41.7.3100-3111.2003 PMC165321

[16] Matthijnssens J, Ciarlet M, McDonald SM, Attoui H, Banyai K, et al (2011) Uniformity of rotavirus strain nomenclature proposed by the Rotavirus Classification Working Group (RCWG). Arch Virol 156: 1397–1413. 21597953 10.1007/s00705-011-1006-z PMC3398998

[17] Santos N, Hoshino Y (2005) Global distribution of rotavirus serotypes/genotypes and its implication for the development and implementation of an effective rotavirus vaccine. Rev Med Virol 15: 29–56. 15484186 10.1002/rmv.448

[18] Cunliffe NA, Ngwira BM, Dove W, Nakagomi O, Nakagomi T, et al (2009) Serotype g12 rotaviruses, Lilongwe, Malawi. Emerg Infect Dis 15: 87–90. 19116060 10.3201/eid1501.080427 PMC2660691

[19] Page NA, de Beer MC, Seheri LM, Dewar JB, Steele AD (2009) The detection and molecular characterization of human G12 genotypes in South Africa. J Med Virol 81: 106–113. 19031449 10.1002/jmv.21362

[20] Oluwatoyin Japhet M, Adeyemi Adesina O, Famurewa O, Svensson L, Nordgren J (2012) Molecular epidemiology of rotavirus and norovirus in Ile-Ife, Nigeria: high prevalence of G12P[8] rotavirus strains and detection of a rare norovirus genotype. J Med Virol 84: 1489–1496. 22825829 10.1002/jmv.23343

[21] Chandrahasen C, Grimwood K, Redshaw N, Rich FJ, Wood C, et al (2010) Geographical differences in the proportion of human group A rotavirus strains within New Zealand during one epidemic season. Journal of Medical Virology 82: 897–902. 20336723 10.1002/jmv.21739

[22] Esona MD, Geyer A, Page N, Trabelsi A, Fodha I, et al (2009) Genomic characterization of human rotavirus G8 strains from the African rotavirus network: relationship to animal rotaviruses. J Med Virol 81: 937–951. 19319943 10.1002/jmv.21468

[23] Esona MD, Steele D, Kerin T, Armah G, Peenze I, et al (2010) Determination of the G and P Types of Previously Nontypeable Rotavirus Strains from the African Rotavirus Network, 1996–2004: Identification of Unusual G Types. Journal of Infectious Diseases 202: S49–S54. 20684717 10.1086/653552

[24] Gentsch JR, Laird AR, Bielfelt B, Griffin DD, Bányai K, et al (2005) Serotype Diversity and Reassortment between Human and Animal Rotavirus Strains: Implications for Rotavirus Vaccine Programs. Journal of Infectious Diseases 192: S146–S159. 16088798 10.1086/431499

[25] Kiulia NM, Kamenwa R, Irimu G, Nyangao JO, Gatheru Z, et al (2008) The epidemiology of human rotavirus associated with diarrhoea in Kenyan children: a review. J Trop Pediatr 54: 401–405. 18593738 10.1093/tropej/fmn052

[26] Matthijnssens J, Rahman M, Yang X, Delbeke T, Arijs I, et al (2006) G8 Rotavirus Strains Isolated in the Democratic Republic of Congo Belong to the DS-1-Like Genogroup. Journal of Clinical Microbiology 44: 1801–1809. 16672410 10.1128/JCM.44.5.1801-1809.2006 PMC1479174

[27] Pietsch C, Petersen L, Patzer L, Liebert UG (2009) Molecular Characteristics of German G8P[4] Rotavirus Strain GER1H-09 Suggest that a Genotyping and Subclassification Update Is Required for G8. Journal of Clinical Microbiology 47: 3569–3576. 19741083 10.1128/JCM.01471-09 PMC2772587

[28] Steyer A, Poljšak-Prijatelj M, Bufon TL, Marčun-Varda N, Marin J (2007) Rotavirus genotypes in Slovenia: Unexpected detection of G8P[8] and G12P[8] genotypes. Journal of Medical Virology 79: 626–632. 17387749 10.1002/jmv.20811

[29] Levy K, Hubbard AE, Eisenberg JN (2009) Seasonality of rotavirus disease in the tropics: a systematic review and meta-analysis. Int J Epidemiol 38: 1487–1496. 19056806 10.1093/ije/dyn260 PMC2800782

[30] Cook SM, Glass RI, LeBaron CW, Ho MS (1990) Global seasonality of rotavirus infections. Bull World Health Organ 68: 171–177. 1694734 PMC2393128

[31] Pitzer VE, Viboud C, Simonsen L, Steiner C, Panozzo CA, et al (2009) Demographic variability, vaccination, and the spatiotemporal dynamics of rotavirus epidemics. Science 325: 290–294. 19608910 10.1126/science.1172330 PMC3010406

[32] D'Souza RM, Hall G, Becker NG (2008) Climatic factors associated with hospitalizations for rotavirus diarrhoea in children under 5 years of age. Epidemiol Infect 136: 56–64. 17352836 10.1017/S0950268807008229 PMC2870768

[33] Atchison CJ, Tam CC, Hajat S, van Pelt W, Cowden JM, et al (2010) Temperature-dependent transmission of rotavirus in Great Britain and The Netherlands. Proc Biol Sci 277: 933–942. 19939844 10.1098/rspb.2009.1755 PMC2842727

[34] Jagai JS, Sarkar R, Castronovo D, Kattula D, McEntee J, et al (2012) Seasonality of rotavirus in South Asia: a meta-analysis approach assessing associations with temperature, precipitation, and vegetation index. PLoS One 7: e38168. 22693594 10.1371/journal.pone.0038168 PMC3364973

[35] PATH (2012) Vaccine Access and Delivery Global Program Rotavirus disease and vaccines in Tanzania. Seattle USA.

[36] Moyo SJ, Gro N, Kirsti V, Matee MI, Kitundu J, et al (2007) Prevalence of enteropathogenic viruses and molecular characterization of group A rotavirus among children with diarrhea in Dar es Salaam Tanzania. BMC Public Health 7: 359. 18162127 10.1186/1471-2458-7-359 PMC2235852

[37] Ezeamama AE, Spiegelman D, Hertzmark E, Bosch RJ, Manji KP, et al (2012) HIV infection and the incidence of malaria among HIV-exposed children from Tanzania. J Infect Dis 205: 1486–1494. 22457274 10.1093/infdis/jis234 PMC3415816

[38] WHO (1977) The presentation and use of height and weight data for comparing the nutritional status of groups of children under the age of 10 years. Bull World Health Organ 55: 489–498. 304391 PMC2366685

[39] EUROROTA (2009) European Rotavirus Detection and Typing Methods.

[40] Kimura M (1980) A simple method for estimating evolutionary rates of base substitutions through comparative studies of nucleotide sequences. J Mol Evol 16: 111–120. 7463489 10.1007/BF01731581

[41] Brookfield DSK, Cosgrove BP, Bell EJ, Madeley CR (1979) Viruses demonstrated in children in Tanzania: studies in diarrhoea and measles. Journal of Infection 1: 249–255.

[42] Sam NE, Haukenes G, Szilvay AM, Mhalu F (1992) Rotavirus infection in Tanzania: a virological, epidemiological and clinical study among young children. APMIS 100: 790–796. 1327004 10.1111/j.1699-0463.1992.tb04001.x

[43] Mhalu FS, Myrmel H, Msengi A, Haukenes G (1988) Prevalence of infection with rotavirus and enteric adenoviruses among children in Tanzania. NIPH Ann 11: 3–7. 2841626

[44] Aminu M, Page NA, Ahmad AA, Umoh JU, Dewar J, et al (2010) Diversity of rotavirus VP7 and VP4 genotypes in Northwestern Nigeria. J Infect Dis 202 Suppl: S198–204 10.1086/653570 20684703

[45] Arora R, Chitambar SD (2011) Full genomic analysis of Indian G1P[8] rotavirus strains. Infect Genet Evol 11: 504–511. 21256248 10.1016/j.meegid.2011.01.005

[46] Aladin F, Nawaz S, Iturriza-Gomara M, Gray J (2010) Identification of G8 rotavirus strains determined as G12 by rotavirus genotyping PCR: updating the current genotyping methods. J Clin Virol 47: 340–344. 20138804 10.1016/j.jcv.2010.01.004

[47] Iturriza-Gomara M, Kang G, Gray J (2004) Rotavirus genotyping: keeping up with an evolving population of human rotaviruses. J Clin Virol 31: 259–265. 15494266 10.1016/j.jcv.2004.04.009

[48] Nordgren J, Nitiema LW, Sharma S, Ouermi D, Traore AS, et al (2012) Emergence of unusual G6P[6] rotaviruses in children, Burkina Faso, 2009–2010. Emerg Infect Dis 18: 589–597. 22469076 10.3201/eid1804.110973 PMC3309693

[49] Cortese MM, Parashar UD (2009) Centers for Disease Control and Prevention (2009) Prevention of rotavirus gastroenteritis among infants and children: recommendations of the Advisory Committee on Immunization Practices (ACIP). MMWR Recomm Rep 58: 1–25. 19194371

[50] Afrad MH, Hassan Z, Farjana S, Moni S, Barua S, et al (2013) Changing profile of rotavirus genotypes in Bangladesh, 2006–2012. BMC Infect Dis 13: 320. 23855423 10.1186/1471-2334-13-320 PMC3723515

[51] Madhi SA, Cunliffe NA, Steele D, Witte D, Kirsten M, et al (2010) Effect of human rotavirus vaccine on severe diarrhea in African infants. N Engl J Med 362: 289–298. 20107214 10.1056/NEJMoa0904797

[52] Parashar UD, Glass RI (2009) Rotavirus vaccines–early success, remaining questions. N Engl J Med 360: 1063–1065. 19279338 10.1056/NEJMp0810154

[53] Patel M, Shane AL, Parashar UD, Jiang B, Gentsch JR, et al (2009) Oral rotavirus vaccines: how well will they work where they are needed most? J Infect Dis 200 Suppl 1S39–48. 19817613 10.1086/605035 PMC3673012

[54] Cegielski JP, Msengi AE, Miller SE (1994) Enteric viruses associated with HIV infection in Tanzanian children with chronic diarrhea. Pediatr AIDS HIV Infect 5: 296–299. 11361370

[55] Cunliffe NA, Gondwe JS, Kirkwood CD, Graham SM, Nhlane NM, et al (2001) Effect of concomitant HIV infection on presentation and outcome of rotavirus gastroenteritis in Malawian children. Lancet 358: 550–555. 11520526 10.1016/s0140-6736(01)05706-3

[56] Liste MB, Natera I, Suarez JA, Pujol FH, Liprandi F, et al (2000) Enteric virus infections and diarrhea in healthy and human immunodeficiency virus-infected children. J Clin Microbiol 38: 2873–2877. 10921942 10.1128/jcm.38.8.2873-2877.2000 PMC87134

[57] Oshitani H, Kasolo FC, Mpabalwani M, Luo NP, Matsubayashi N, et al (1994) Association of rotavirus and human immunodeficiency virus infection in children hospitalized with acute diarrhea, Lusaka, Zambia. J Infect Dis 169: 897–900. 8133106 10.1093/infdis/169.4.897

[58] Pavia AT, Long EG, Ryder RW, Nsa W, Puhr ND, et al (1992) Diarrhea among African children born to human immunodeficiency virus 1-infected mothers: clinical, microbiologic and epidemiologic features. Pediatr Infect Dis J 11: 996–1003. 1461710 10.1097/00006454-199211120-00002

[59] Rossit AR, de Almeida MT, Nogueira CA, da Costa Oliveira JG, Barbosa DM, et al (2007) Bacterial, yeast, parasitic, and viral enteropathogens in HIV-infected children from Sao Paulo State, Southeastern Brazil. Diagn Microbiol Infect Dis 57: 59–66. 17178297 10.1016/j.diagmicrobio.2006.11.005

[60] Bresee JS, Glass RI, Ivanoff B, Gentsch JR (1999) Current status and future priorities for rotavirus vaccine development, evaluation and implementation in developing countries. Vaccine 17: 2207–2222. 10403588 10.1016/s0264-410x(98)00376-4

